# Differentiating between cancer and normal tissue samples using multi-hit combinations of genetic mutations

**DOI:** 10.1038/s41598-018-37835-6

**Published:** 2019-01-30

**Authors:** Sajal Dash, Nicholas A. Kinney, Robin T. Varghese, Harold R. Garner, Wu-chun Feng, Ramu Anandakrishnan

**Affiliations:** 1 Department of Computer Science, Virginia Tech, Blacksburg, VA USA; 2 Biomedical Sciences, Edward Via College of Osteopathic Medicine, Blacksburg, VA USA; 3 Department of Electrical and Computer Engineering, Virginia Tech, Blacksburg, VA USA; 4 Gibbs Cancer Center and Research Institute, Spartanburg, SC USA

**Keywords:** Cancer, Computational biology and bioinformatics, Cancer genomics

## Abstract

Cancer is known to result from a combination of a small number of genetic defects. However, the specific combinations of mutations responsible for the vast majority of cancers have not been identified. Current computational approaches focus on identifying driver genes and mutations. Although individually these mutations can increase the risk of cancer they do not result in cancer without additional mutations. We present a fundamentally different approach for identifying the cause of individual instances of cancer: we search for combinations of genes with carcinogenic mutations (multi-hit combinations) instead of individual driver genes or mutations. We developed an algorithm that identified a set of multi-hit combinations that differentiate between tumor and normal tissue samples with 91% sensitivity (95% Confidence Interval (CI) = 89–92%) and 93% specificity (95% CI = 91–94%) on average for seventeen cancer types. We then present an approach based on mutational profile that can be used to distinguish between driver and passenger mutations within these genes. These combinations, with experimental validation, can aid in better diagnosis, provide insights into the etiology of cancer, and provide a rational basis for designing targeted combination therapies.

## Introduction

Experimental studies and mathematical models suggest that carcinogenesis is likely a result of different combinations of a small number of carcinogenic mutations (hits)^1–7^. Mathematical models estimate that the number of such hits varies from two to eight^1–8^. Yet, our collective computational and experimental efforts and the accumulation of cancer genomic data have failed to identify, for most cancers, the specific combinations of mutations triggering carcinogenesis.

Current computational efforts to find carcinogenic mutations generally focus on identifying individual “driver mutations”, based on mutational frequency and signatures^9–12^. These driver mutations have been shown to be associated with an increased risk of cancer. However, they can not generally cause cancer by themselves. For example, 72% of women with an inherited BRCA1 mutation are likely to get cancer by age 80. However, even for women with the BRCA1 mutation, none are likely to get cancer before age 20, and 28% of them may never get cancer^13^. The Li Fraumeni syndrome is another example where germline P53 mutations is associated with early onset cancer predisposition (e.g. soft tissue and bone sarcomas). However, cancer penetrance is less than 20% for children while approaching 80% by age 70, indicating that multiple hits are required for carcinogenesis^14–17^. The relationship between most other known genetic markers and increased cancer risk is far weaker^18,19^. The limited early cancer incidence in individuals with germline mutations suggests that additional genetic defects acquired over an individual’s lifetime are necessary for carcinogenesis. Therefore, current computational approaches focused on identifying individual genes that are cancer drivers, cannot find the specific combinations of mutations responsible for individual instances of cancer. Several factors, other than genetic mutations, have also been implicated in carcinogenesis, such as epigenetic modifications^20^, tumor environment^21^, and adaptive evolution^22^. However, carcinogenesis is primarily a result of genetic mutations^23^.

The goal of this work is to develop a method for identifying combinations of genetic mutations that are most likely responsible for individual instances of cancer. This goal is fundamentally different from identifying the most frequent driver mutations, and represents the first computational study to specifically identify multi-hit combinations. Our approach consists of first identifying likely combinations of genes with carcinogenic mutations. We then present a method, based on the mutational profile of these genes, for identifying likely carcinogenic mutations within these genes. Although it is theoretically possible to search for combinations of individual mutations using our method, the problem becomes computationally intractable, since most genes contain hundreds of somatic mutations. In addition, in the much larger set of somatic mutation combinations many carcinogenic combinations will be rarely represented, further increasing the challenge of identifying these combinations. Therefore, we chose to first identify combinations of genes with somatic mutations, and then present an approach for identifying likely carcinogenic mutations within these genes.

We mapped the problem of finding these combinations to the extensively studied weighted set cover (WSC) problem^24^. Finding the optimal solution to the corresponding WSC problem is computationally intractable due to the exponentially large number of possible sets of multi-hit combinations. However, there exist approximation algorithms for finding near-optimal solutions^24,25^. We adapted one such algorithm to find a set of multi-hit combinations that maximizes the number of tumor samples that contain one of these multi-hit combinations while minimizing the number of normal samples that contain any of these combinations. The number of candidate set covers is an exponentially large quantity due to the large number of possible combinations. We applied the above algorithm to find a set of 2-hit combinations using somatic mutation data from the cancer genome atlas (TCGA). For the 17 cancer types with at least 200 matched tumor and blood-derived normal samples in TCGA, the algorithm identified a set of 197 2-hit combinations. For a separate set of Test samples, these combinations were able to differentiate between tumor and normal samples with 91% sensitivity (95% Confidence Interval (CI) = 89–92%) and 93% specificity (95% CI = 91–94%) on average, for the 17 cancer types. The results are consistent across different randomly selected Training and Test sets. Despite this high accuracy, our analysis of the results shows that many of the 2-hit combinations are likely to be two-gene subsets of three or more-gene combinations. We discuss how carcinogenic and non-carcinogenic mutations within the gene combinations can be distinguished. We also discuss how the multi-hit combinations can be used to develop targeted combination therapy.

Identifying gene combinations is important for two reasons. First, it brings us closer to the understanding of carcinogenesis and the complexity of cancer biology. Second, the identification of the specific combination responsible for a given instance of cancer can help us design more effective combination therapies for treating the disease. Combination therapies can be more effective than single target treatments; however, most current therapeutic combinations have been based on trial and error^26,27^. Identifying the precise combination of genomic anomalies responsible for individual instances of cancer provides a more rational basis for designing combination therapies.

In the Methods section, we present our approach for finding genes with mutations responsible for cancer. We describe the mapping of the problem to the weighted set cover (WSC) problem and the WSC approximation algorithm used to identify the multi-hit combinations. In the Results section, we show that our approach can identify a set of multi-hit combinations that can differentiate between tumor tissue and normal tissue samples with over 90% sensitivity and specificity. This result is robust to different randomly selected training and test sets. We discuss how these combinations can be further analyzed to distinguish carcinogenic and non-carcinogenic mutations within genes and how they may be used to design targeted combination therapies.

## Results

We implemented a weighted set cover algorithm to identify 2-hit combinations of cancer causing genes with mutations using a randomly selected Training set of tumor and normal tissue samples (see *Methods*). The set of combinations distinguish between tumor and normal tissue samples with over 90% sensitivity and specificity. This result is robust to different Training and Test set partitions of the available tumor and normal tissue samples. Although the identified combinations contain many genes previously implicated in cancer, our approach has also identified several potentially novel cancer genes. Our results suggest that some of the combinations identified are 2-hit subsets of 3+ hit combinations.

### A set of 2-hit combinations can differentiate between tumor and normal tissue samples with high accuracy

We implemented the weighted set cover algorithm described in *Methods*, for identifying a set of 2-hit combinations with the goal of maximizing accuracy (sensitivity and specificity) in differentiating between tumor and normal samples. Using a randomly selected Training set (see *Methods*), we identified a set of 2-hit combinations for each of the seventeen cancer types with at least two hundred matched tumor and blood-derived normal samples.

When tested against a separate randomly selected Test set, the identified set of combinations were able to differentiate between tumor tissue samples and normal tissue samples, for their respective cancer types, with greater than 90% specificity and sensitivity on average. Table 1 shows the sample sizes, sensitivity, and specificity for the Training and Test sets for each of the seventeen cancer types. Sensitivity varies from 83% to 100% and specificity varies from 86% to 100%, depending on cancer type.

**Table 1:** 2-hit combinations can differentiate between tumor and normal tissue samples with over 90% sensitivity and specificity.

		Training Set								Test Set									
		Tumor Samples				Normal Samples				Tumor Samples					Normal Samples				
Cancer Type	#Comb-inations	True Positives	False Negatives	Total	Sensi-tivity	True Negatives	False Positives	Total	Speci-ficity	True Positives	False Negatives	Total	Sensi-tivity	95% CI	True Negatives	False Positives	Total	Speci-ficity	95% CI
Bladder Urothelial Carcinoma (BLCA)	18	267	0	267	100%	245	2	247	99%	89	12	101	88%	80–93%	74	12	86	86%	76–92%
Breast invasive carcinoma (BRCA)	8	703	0	703	100%	236	11	247	96%	207	1	208	100%	97–99%	82	4	86	95%	88–98%
Cervical squamous cell carcinoma and endocervical adenocarcinoma (CESC)	9	217	0	217	100%	247	0	247	100%	52	5	57	91%	80–97%	84	2	86	98%	91–99%
Colon adenocarcinoma (COAD)	9	291	0	291	100%	245	2	247	99%	85	9	94	90%	82–95%	83	3	86	97%	90–99%
Glioblastoma multiforme (GBM)	10	253	0	253	100%	247	0	247	100%	72	6	78	92%	84–97%	78	8	86	91%	82–95%
Head and Neck squamous cell carcinoma (HNSC)	13	347	0	347	100%	245	2	247	99%	102	21	123	83%	75–89%	81	5	86	94%	86–98%
Kidney renal papillary cell carcinoma (KIRP)	11	175	0	175	100%	246	1	247	100%	50	3	53	94%	84–98%	86	0	86	100%	95–100%
Brain Lower Grade Glioma (LGG)	9	356	0	356	100%	245	2	247	99%	111	12	123	90%	83–94%	80	6	86	93%	85–97%
Liver hepatocellular carcinoma (LIHC)	9	233	0	233	100%	246	1	247	100%	78	1	79	99%	93–99%	79	7	86	92%	83–96%
Lung adenocarcinoma (LUAD)	13	318	0	318	100%	245	2	247	99%	83	8	91	91%	83–96%	79	7	86	92%	83–96%
Lung squamous cell carcinoma (LUSC)	12	224	0	224	100%	246	1	247	100%	68	13	81	84%	74–91%	82	4	86	95%	88–98%
Ovarian serous cystadeno-carcinoma (OV)	8	235	0	235	100%	246	1	247	100%	75	7	82	91%	83–96%	83	3	86	97%	90–99%
Prostate adenocarcinoma (PRAD)	20	327	0	327	100%	245	2	247	99%	83	11	94	88%	80–94%	68	18	86	79%	68–87%
Sarcoma (SARC)	6	167	0	167	100%	247	0	247	100%	47	5	52	90%	78–96%	86	0	86	1.00	95–100%
Stomach adenocarcinoma (STAD)	19	306	0	306	100%	247	0	247	100%	72	10	82	88%	78–93%	77	9	86	90%	81–95%
Thyroid carcinoma (THCA)	13	314	0	314	100%	245	2	247	99%	94	13	107	88%	80–93%	78	8	86	91%	82–95%
Uterine Corpus Endometrial Carcinoma (UCEC)	10	368	0	368	100%	247	0	247	100%	121	6	127	95%	90–98%	81	5	86	94%	86–98%
Total	197	5101	0	5101	100%	4170	29	4199	99%	1489	143	1632	91%	89–92%	1361	101	1462	93%	91–94%

The combinations were identified using a randomly selected 75% subset (Training set) of the available matched tumor and blood-derived normal samples for each cancer type with at least 200 matched samples in TCGA. See Tables S2–S18 for the list of gene combinations for each cancer type. The resulting combinations were then tested against the remaining samples (Test set).

**Figure 1:**
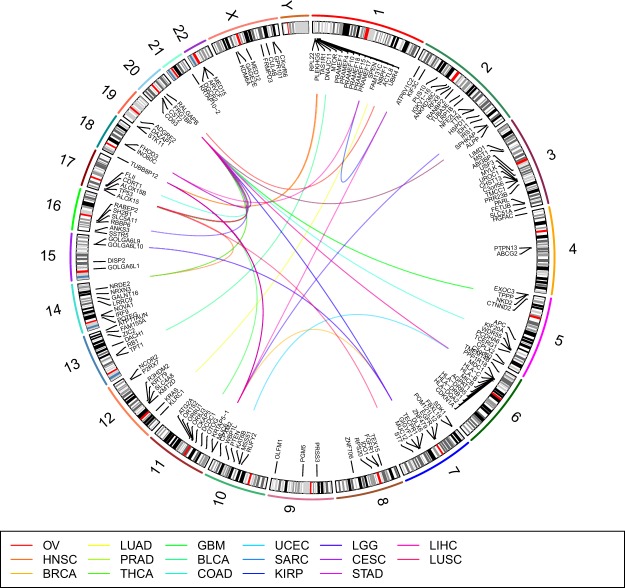
Top three 2-hit combinations for 17 cancer types. See Table 1 for abbreviations for cancer types. Each line in the center of the Circos plot connects the two genes in a 2-hit combination. This plot was generated using RCircos^43^.

The number of combinations identified varies from 8–20 for the 17 cancer types (Table 1). In total, 197 combinations were identified (Tables S2–S18). The top three 2-hit combinations are summarized in Fig. 1. The combinations include 256 unique genes with 138 genes occurring in more than one combination.

### Results are robust to different Training and Test sets

**Figure 2:**
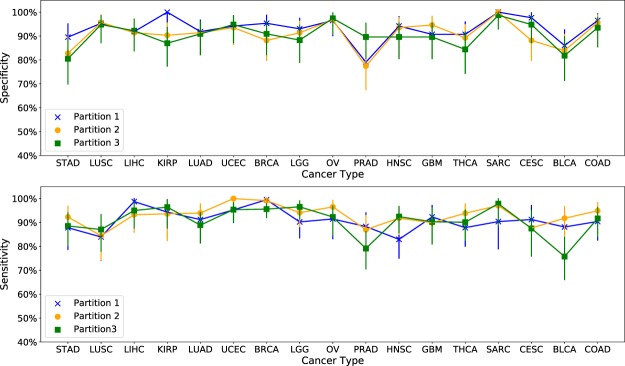
Sensitivity and specificity is robust across three different random training-test partitions of available samples. The average difference between any two pairs of partitionings is less than 4.2% for both sensitivity and specificity across all seventeen cancer types. Error bars represent 95% confidence intervals.

**Figure 3:**
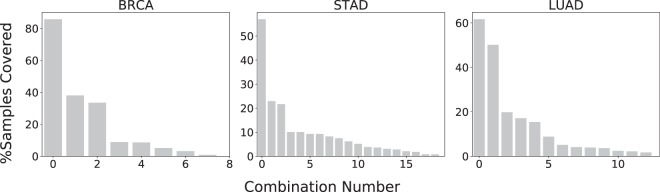
Occurrence of the 2-hit combinations identified in tumor samples, for three representative cancer types. Figure S3 shows the distribution for all seventeen cancer types. The top combination occurs in 65% of tumor samples, on average, while 42% of the combinations occur in less than 5% of the samples. Total percentage exceeds 100% because samples can contain multiple combinations.

To test the robustness of the above results, we randomly re-partitioned the available samples into two more alternative Training and Test sets. Figure 2 shows specificity and sensitivity of the algorithm across the seventeen cancer types considered here, for three different sets of partitions. The average difference in sensitivity between any two pairs of train-test partitions is less than 4.2% and the average difference in specificity is less than 4.1%. The largest difference in sensitivity is 12% (BLCA) and the largest difference in specificity is 13% (KIRP). In addition, the most frequently occurring combinations in the tumor samples were the same between any two train-test partitions for 14 of 17 cancer types, representing 65% of tumor samples (Fig. 3). However, there were significant differences between the less frequently occurring combinations with only 39 common combinations, out of 197 total combinations, across the three sets of combinations for the three training-test partitions  (Fig. S2). Clearly, the samples included in the Training set affect the set of combinations identified. This is to be expected since 42% of the combinations occur in less than 5% of the samples for each cancer type (Fig. S4). Different partitions of the tumor samples will result in different sets of these rare combinations being included in the Training set, resulting in different combinations being identified. In addition, since the approximation algorithm used here identifies a near-optimal solution, changes in the Training set can result in different near-optimal combinations being selected by the algorithm.

### The combinations identified include novel cancer genes

The genes comprising the 2-hit combinations identified above fall into three categories. (1) Confirmed cancer genes based on the Catalog of Somatic Mutations in Cancer (COSMIC) database^28^. (2) Non-COSMIC genes that have been implicated in cancer based on experimental evidence. (3) Genes that have not been experimentally implicated in cancer. Table 2 summarizes, from Tables S2–S18, the 31 genes that comprise the top three most frequently occurring 2-hit combinations for each of the cancer types studied. Of these genes, nine are confirmed cancer genes (e.g. APC, IDH1, KRAS, PTEN, RB1, and TP53), thirteen have been experimentally implicated in cancer (e.g. HLA-C, IGHG1, and KCNB1), and nine have not previously been implicated in cancer (e.g. TUBBP12).

**Table 2:** Genes in the top three most frequently occurring 2-hit combinations.

Gene	Cancer Type																
	BLCA	BRCA	CESC	COAD	GBM	HNSC	KIRP	LGG	LIHC	LUAD	LUSC	OV	PRAD	SARC	STAD	THCA	UCEC
**Confirmed Cancer Genes - in COSMIC**																	
APC^46^				**1**													
CTNND2^47^	**2**												**2**			**2**	
IDH1^48^								**1**									
KRAS^49^										**3**							
MUC12^50^		**1**	**2,3**											**1,3**			**1**
MUC6^51^	**1**	**1,3**	**1**	**2**		**1**	**1**	**1,2**	**2**	**1**	**1**	**1**	**1**	**2**	**1**	**1**	**2**
PTEN^52^																	**3**
RB1^53^	**3**																
TP53^54^						**2**					**2,3**	**2**					
**Experimentally Implicated in Cancer**																	
ALOX15^55^					**3**												
ALPP^56^													**3**		**3**	**3**	
CACNA1E^57^										**3**							
CCDC30^58^	**3**																
DPP6^59^											**3**						
FHOD3^60^							**2**		**1,3**								
FRG1BP^61^	**2**	**2**	**2**	**1,3**	**2,3**	**3**	**2**	**3**	**1**	**2**	**2**	**2**	**2,3**	**1**	**2,3**	**2,3**	**1**
HLA-C^62^						**3**									**2**		
HLA-DRB1^63^																	**3**
HRNR^64^							**3**										
IGHG1^38^		**2**			**2**	**2**				**2**							
KCNB1^65^												**1**					
NBPF1^66^		**3**															
SLC5A11^67^								**3**									
**Potentially Novel Cancer Genes**																	
CCDC43				**3**													
GOLGA6L10			**3**														
GOLGA6L9														**3**			
LCE1A									**3**								
OR2T7							**3**		**2**								
OR8U1					**1**												
PRAMF15												**3**					
TUBB8P12	**1**		**1**	**2**	**1**	**1**	**1**	**2**		**1**	**1**	**3**	**1**	**2**	**1**	**1**	**2**

Genes are grouped by those that are confirmed cancer genes, experimentally implicated in cancer, and potentially novel cancer genes. The numbers in the table (1, 2, and/or 3) indicate which of the top three 2-hit combinations the gene belongs to.

The genes in the last category have not been extensively studied, and represent potentially novel cancer genes. For example, TUBB8P12 (Tubulin Beta 8 Pseudogene 12) occurs in the top three 2-hit combinations in 15 of the 17 cancer types. However, TUBB8P12 has not been previously identified as frequently mutated in cancers. There are two possible reasons why we have identified TUBB8P12 as a potential cancer gene while previous bioinformatics studies have not. The first reason is that, we considered low frequency somatic mutations, identified using matched tumor and blood derived normal samples, that were not included in many of the previous studies^9,12,29,30^. Biopsy specimens contain a mix of tumor and normal tissue cells, tumor infiltrating lymphocytes, and stromal cells. In addition, tumor cells themselves can be genetically diverse. Therefore many somatic mutations are likely to be present at very low frequencies^30,31^. Studies that use masked open-access TCGA data will exclude many such low-frequency mutations. The second reason is that, those studies that do use controlled-access TCGA data that include these low-frequency mutations, do not use matched normal tissue and blood-derived normal samples to quantify the differential mutation frequency between tumor and normal samples^9–12^. By comparing somatic mutation frequency in matched tumor tissue samples to mutation frequency in matched normal tissue samples, we are able to identify genes that are significantly more frequently mutated in tumor samples relative to normal samples, while excluding genes that may be highly mutated in both tumor and normal samples.

### The 2-hit combinations may represent subsets of a larger number of hits

**Figure 4:**
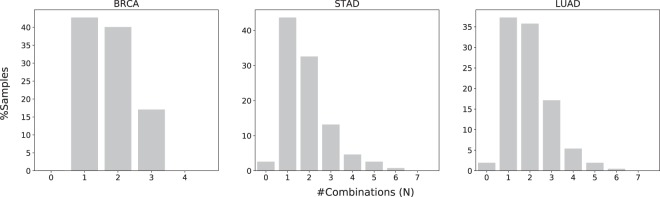
Distribution of overlapping combinations for three representative cancer types. Figure S5 shows the distribution for all seventeen cancer types. 64.5% tumor samples contain multiple combinations, suggesting that the 2-hit combinations might represent subsets of three or more hits.

Due to practical limitations of computational resources, it is not practical to search for more than 2-hit combinations using the current version of the algorithm presented (see *Methods*). The computer run times for identifying 2-hit combinations were ≈2 hours, compared to estimated run times of over 1 year for 3-hit combinations. Mathematical models predict that the likely number of hits required for carcinogenesis ranges from two to eight. Therefore, it is likely that the 2-hit combinations identified here are different subsets of three or more hits In fact, we find that 65% of the samples contain multiple combinations (Fig. 4), and 138 of the 256 genes in these combinations occur in more than one combination, suggesting that the genes in the different 2-hit combinations within a sample may instead represent a single combination consisting of more than 2-hits. Therefore, the two hit combinations may produce some false positives in normal samples containing mutations in only two genes of a 3+ hit combination. Therefore, searching for three or more hits may further improve the accuracy of our results.

### Genes within combinations are not correlated

**Figure 5:**
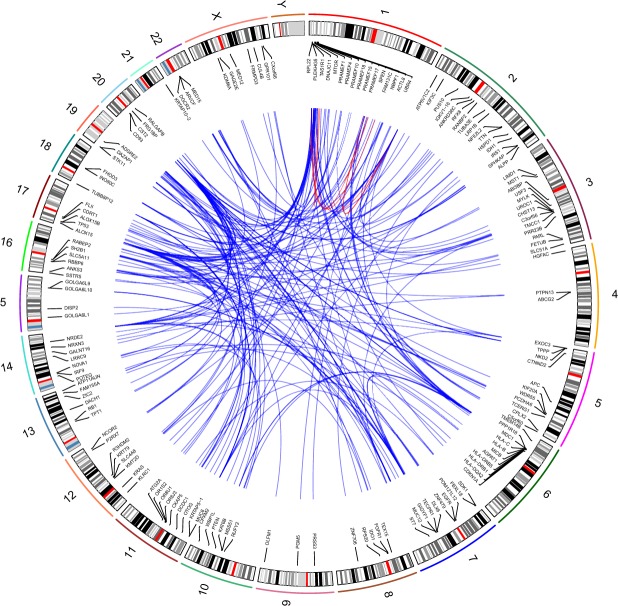
Chromosomal location of gene combinations. Each connecting line represents a 2-hit combination. Blue lines represent gene combinations across different chromosomes. Red lines represent gene combinations within the same chromosome. Circos plot was generated using RCircos^43^.

Analysis of genes within each combination shows that they are not correlated. For each of the genes in a combination we construct a vector of 0’s and 1’s. The length of the vector is equal to the number of normal samples, and the value in the *i*^*th*^ position of that vector represents whether the *i*^*th*^ normal sample has a protein-altering mutation (as determined by the Variant Effect Predictor (VEP)) in that location or not. Then we computed Pearson’s correlation coefficient^32^ using stats. pearsonr routine from python module scipy. stats between two vectors representing two different genes. The Pearson correlation coefficient is less than 0.25 for the gene pairs within each combination (Fig. S1). If the genes within a combination were correlated it would have suggested that the combination is a result of some common underlying cause, such as being a passenger mutation or due to structural chromosomal modification, and unlikely to be causative. We also examined the chromosomal location of genes within each combination (Fig. 5). Only two of the 197 combinations contain genes within the same chromosome, suggesting that the genes within combinations are not due to a chromosomal abnormality that may affect multiple genes within a chromosome.

## Discussion

Here we discuss how the multi-hit combinations identified above can be used to identify carcinogenic (driver) and non-carcinogenic (passenger) mutations within genes. We also illustrate how these combinations may be used to design a combination therapy targeting the specific genetic mutations responsible for individual instances of cancer.

### Distinguishing between driver and passenger mutations

The method used to identify multi-hit combinations uses a mutation frequency based approach to preferentially select driver genes instead of passenger genes, i.e. the selected genes have a significantly higher mutation frequency in tumor samples compared to normal samples. For each gene, the mutation frequency in normal samples is considered to be approximately representative of the background mutation frequency for the gene. However, within these genes not all mutations are carcinogenic.

**Figure 6:**
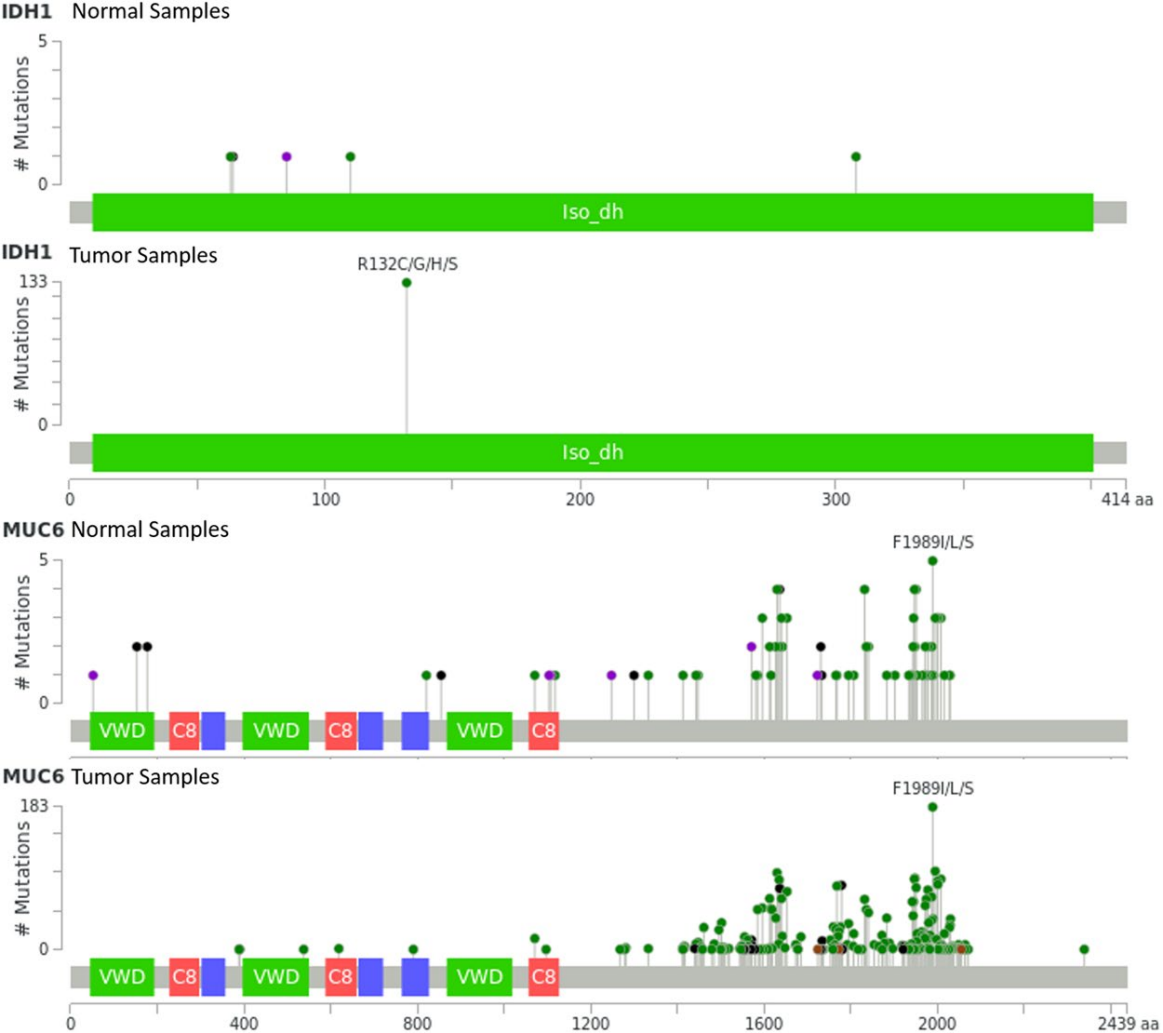
Mutations in normal and lower grade glioma (LGG) tumor samples with mutations in both IDH1 and MUC6. The difference in mutations between normal and tumor samples for the same 2-hit combination can be used to further refine the search algorithm. In the above examples, a missense mutation at R132 in IDH1 is likely to be carcinogenic, whereas mutations at F1989 in MUC6 are unlikely to be carcinogenic. Colored bars represent known functional protein domains. Grey bars represent regions of unknown function. Green dots represent missense mutations, black dots represent truncating mutations and purple dots represent other protein-altering mutations. Figure generated using cBioPortal (Cerami *et al*. and Gao *et al*.)^44,45^.

The combinations found above provide a starting point for examining a smaller subset of genes more closely to identify specific carcinogenic mutations within these genes. In identifying the multi-hit combinations, we did not take into consideration the location of mutations within genes. Clearly there are locations within a gene where certain mutations are unlikely to affect the function of the gene product. Such mutations can result in false positives and contribute to the large number (65%) of tumor samples containing multiple combinations (Fig. 4). Consider for example, the 2-hit combination of mutations in IDH1 and MUC6 in brain lower grade glioma (LGG) tumor samples. Of the 479 LGG tumor samples, 134 (28%) contain mutations in both IDH1 and MUC6, while 5 (1.5%) of 333 normal tissue samples contain a mutation in both these genes (Fig. 6). Comparing the mutations within these genes for normal and tumor samples may reveal which are carcinogenic and which are not. In this example, every one of the tumor samples contains a missense mutation at R132 in IDH1 and no other mutations, while the normal samples do not contain any mutations at this position (Fig. 6). Mutations at R132 in IDH1 have previously been implicated in cancer^33^. On the other hand, the IDH1 mutations seen in the normal samples are unlikely to be carcinogenic. Similarly, mutations at F1989 of MUC6, which occur most frequently in both tumor and normal samples are unlikely to be carcinogenic (Fig. 6). Excluding such non-carcinogenic mutations can reduce the number of false positives and further increase accuracy of our algorithm. In our future work we will develop an automated method to compare and contrast the individual gene loci, so that all of these mutations within genes can be identified. To further improve accuracy of our algorithm, variants that are likely to be carcinogenic can be weighted higher than those that are unlikely to be carcinogenic.

Some of the genes identified by our approach may not be causative (passenger mutations) even though they may be correlated to cancer incidence. Functional analysis can be used to identify genes in the above set of combinations that are unlikely to be driver genes, even though they may be frequently mutated in tumors^11,34,35^. For example, the affect of specific mutations on gene expression levels can be analyzed to determine if the mutation is likely to have a functional effect. In addition we can analyze the pathways affected by the gene combinations (Tables S19–S22). Studies show that combinations of driver gene mutations generally affect mutually exclusive pathways^36^. Therefore, one of the genes in a multi-hit combination affecting the same pathway may include passenger mutations. Although in most cases multiple different pathways are affected by the gene combinations, Tables S19–S22 shows that in some cases (e.g. MUC6 and MUC12 in BRCA) the same pathway is affected by both genes in the combination. Further analysis would be required to determine if the mutations within one of these genes are passenger mutations.

The search algorithm can be run iteratively to incrementally refine the list of multi-hit combinations by excluding these passenger mutations. The input to our algorithm is a list of genes with mutations for each sample. Genes with only passenger mutations can be excluded from this list to minimize the inclusion of passenger mutations in the resulting multi-hit combinations.

### A rational basis for combination therapy

The combinations identified above, with further refinement and clinical validation, may represent a more rational basis for targeted combination therapy, instead of the current “marriages of convenience”^27^ with limited biological rationale^26^. A more rational strategy may also reduce the risk of expensive failures such as the phase III trial of imfinzi plus tremelimumab. The combination of therapies for a given patient could be designed to target specific carcinogenic combinations of gene mutations found in the patient. Although only 30 of the 256 genes in the combinations identified above were formally identified as “cancer genes” in the catalog of somatic mutations in cancer (COSMIC), many of the other genes were previously implicated in cancer (Table 2). Therapies that target many of the genes in both these categories may be available or under development. For example, the combination of mutations in TP53 and IGHG1 occur in 41% of HNSC tumor samples in TCGA. Several drugs that can restore TP53 function, deplete mutant TP53 or affect downstream targets are currently in pre-clinical development^37^. siRNA targeted silencing of IGHG1 has been shown to inhibit cell viability and promote apoptosis, which might therefore act as a potential target in cancer gene therapy^38,39^. For patients with this combination of mutations, a combination therapy targeting both these genes may be more effective in combination, than separately.

## Conclusions

Cancer is many different diseases, although the symptoms may be similar. These different diseases are a result of different combinations of genetic defects (hits). In this study we have developed a method for identifying combinations of genes with mutations that may be responsible for different instances of cancer. Our method is fundamentally different from current approaches which identify individual genes, instead of combinations of genes, in which mutations increase the likelihood of carcinogenesis.

The problem of identifying a set of multi-hit combinations that can differentiate between tumor and normal samples was mapped to the extensively studied weighted set cover (WSC) problem. We adapted a WSC algorithm to the problem of identifying multi-hit combinations. The algorithm was applied to a training set of somatic mutation data from the cancer genome atlas (TCGA) to identify a set of 2-hit combinations for the 17 cancer types with at least 200 matched tumor tissue and blood-derived normal samples. The resulting 2-hit combinations were able to differentiate between tumor and normal tissue samples in a separate test set with over 90% sensitivity and specificity on average. Accuracy of the results were robust to different random partitionings of the available data between training and test sets. The resulting set of combinations include potential novel cancer genes, not previously implicated in cancer.

We show how carcinogenic and non-carcinogenic mutations within genes could be identified, by comparing the occurrence of different mutations in tumor and normal samples. We also illustrate how the combination of mutations responsible for an individual instance of cancer can be used to design a combination therapy targeting the specific genes responsible for that instance of cancer.

## Methods

**Figure 7:**
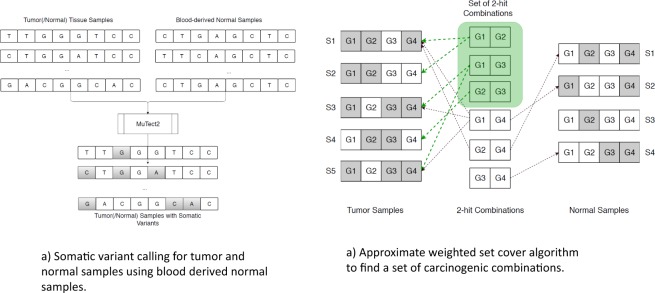
Approach for identifying multi-hit combinations. (**a**) Whole exome sequencing data from The Cancer Genome Atlas (TCGA) for tumor samples and normal tissue samples with matched blood derived normal samples were used to identify somatic mutations. Somatic mutations were calculated using the Mutect2 variant caller and the Variant Effect Predictor (VEP). (**b**) The problem of identifying multi-hit combinations is mapped to the weighted set cover (WSC) problem. An approximate WSC algorithm was used to identify a set of multi-hit combinations that was able to differentiate between an independent set of tumor and normal tissue samples with over 90% sensitivity and specificity.

Our approach for identifying sets of multi-hit combinations consists of two steps (Fig. 7). First, we identified somatic mutations from whole exome sequencing data for tumor and normal tissues with matched blood-derived normal samples from The Cancer Genome Atlas (TCGA). Somatic variants called from matched tumor tissue and blood-derived normal samples can detect low-frequency variants, which would not be detected when using tumor samples alone. Second, we use a weighted set cover algorithm to identify multi-hit combinations that can differentiate between tumor and normal samples with high sensitivity and specificity. The problem of identifying a set of multi-hit combinations is computationally intractable; however, there exist algorithms for finding a near-optimal approximate solution. We used a variant of one such algorithm to identify a set of multi-hit combinations for each cancer type, using a randomly selected subset of the available tumor and normal tissue samples (the Training set). The accuracy (sensitivity and specificity) of the resulting multi-hit combinations was evaluated using the remaining tumor and normal tissue samples (the Test set).

### Somatic mutations calculated from the cancer genome atlas (TCGA) data

The primary input to our algorithm is somatic mutation data for tumor and normal tissue samples. TCGA contains a set of such data for tumor tissue samples with matched blood-derived normal samples, in mutation annotation format (MAF) datasets^40^. These somatic mutations were identified using the commonly used and well documented Mutect2 software. For normal tissue samples we identified a set of 333 normal tissue samples with matched blood-derived normal samples. We calculated somatic mutations for these normal tissue samples using the same Mutect2 protocol used for the tumor tissue samples. We use the Variant Effect Predictor (VEP) to determine the location (intron, exon, UTR) and effect of these variants (synonymous, non-synonymous, missense, nonsense). The specific commands and parameters used are included in Supporting Information (SI). In our analysis we only consider protein-altering variants (non-synonymous, nonsense, and insertion/deletions in exons), as predicted by VEP. We found 6733 tumor samples with ~10^7^ pre-calculated protein-altering somatic variants in the MAF files for the 17 cancer types with at least 200 matched tumor and blood-derived normal samples. In addition, we found 333 matched normal tissue samples in TCGA, in which we identified ~10^6^ protein-altering somatic mutations using the Mutect2/VEP protocol detailed in SI.

The algorithm presented below is based on the somatic mutation data described above, which does not include possible germline mutations that may contribute to carcinogenesis. However, carcinogenic germline mutations are in general relatively rare. For example, BRCA1 is one such rare exception where it occurs as a germline mutation in 5–10% of breast and ovarian cancer patients with a BRCA1 mutation^41,42^. However, the other 90–95% of cases with the BRCA1 mutations are somatic variants. Therefore, the following algorithm should still be able to identify mutations in such genes as carcinogenic, although the possible presence of germline mutations may limit the accuracy of the algorithm.

### Mapping the problem of finding multi-hit combinations to a weighted set cover problem

Our goal is to identify a set of multi-hit combinations of gene mutations, such that at least one combination occurs in each tumor sample while minimizing the number of normal samples containing any of the combinations. Identifying this set of carcinogenic multi-hit combinations can be mapped to the extensively studied weighted set cover (WSC) problem. The WSC problem can be described as follows. For a universal set of elements and a collection of wighted subsets of this universal set, find a minimum weight collection of subsets such that all elements of the universal set are covered. The problem of identifying a set of multi-hit combinations that optimally differentiates between tumor and normal samples can be mapped to the WSC problem as follows.

1. Let, $T=\{{t}_{1},{t}_{2},\ldots ,{t}_{{N}_{t}}\}$ be a set of *N*_*t*_ tumor samples, and $N=\{{n}_{1},{n}_{2},\ldots ,{n}_{{N}_{n}}\}$ be a set of *N*_*n*_ normal samples. We consider *T* as the universal set in the WSC problem.
2. Let *C* = {*c*_1_, *c*_2_, …, *c*_*M*_} be a set of *M* possible combinations. We construct a subset for each of these combinations by taking the tumor samples containing that combination. ${T}^{{c}_{i}}$ represents the subset associated with combination *c*_*i*_, i.e. ${T}^{{c}_{i}}=\{{t}_{1}^{{c}_{i}},{t}_{2}^{{c}_{i}},\ldots \}$, where all tumor samples in ${T}^{{c}_{i}}$ contain the combination *c*_*i*_. Union of all the subsets ${T}^{{c}_{i}}$ constructs the universal set *T*.
3. Assign a weight *w*_*i*_ to each combination *c*_*i*_ (subset ${T}^{{c}_{i}}$ in the WSC problem) such that the weight represents the inverse likelihood of the combination being carcinogenic. *w*_*i*_ is described below. Combinations with lower weights have higher likelihood to be carcinogenic.
4. Find a set of combinations ${C}^{\ast }=\{{c}_{1}^{\ast },{c}_{2}^{\ast },\ldots \}$ such that all the samples in *T* are covered and the total weight $W=\sum {w}_{j}^{\ast }$ is minimized.

**Figure 8:**
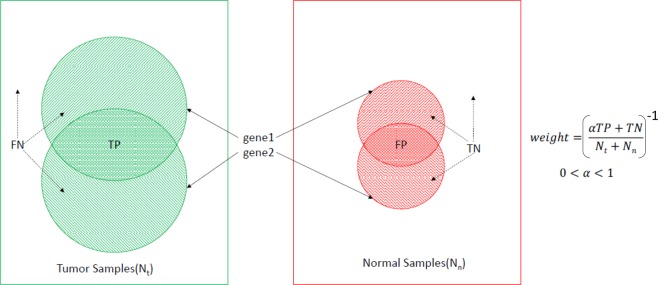
Weight computation for a combination of two genes 〈*gene*1, *gene*2〉. Tumor samples covered by both genes are true positives (TP), tumor samples not covered by one or both genes are false negatives (FN), normal samples covered by both genes are false positives (FP), and normal samples not covered by one or both genes are true negatives (TN). The scaling factor *α* is used to balance the relative importance of sensitivity and specificity.

The goal of the algorithm is to maximize sensitivity *TP*/*N*_*t*_ and specificity *TN*/*N*_*n*_, where *TP* is the number of true positives, *TN* is the number of true negatives, *N*_*t*_ is the number of tumor samples, *N*_*n*_ is the number of normal samples (Fig. 8). Therefore, we assign a weight to each combination as the inverse of the accuracy metric, ${w}_{i}={(\frac{\alpha TP+TN}{{N}_{t}+{N}_{n}})}^{-1}$, where 0 ≤ α ≤ 1 is a scaling factor. The scaling factor is used to balance the optimization of sensitivity and specificity simultaneously. We use the scaling factor 0.1 to reflect the fact that the WSC solution for the Training set always has a true positive rate of 1.0, i.e. every tumor sample in the Training set contains at least one combination.

### Algorithm for finding an approximate solution to the weighted set cover problem

**Figure 9:**
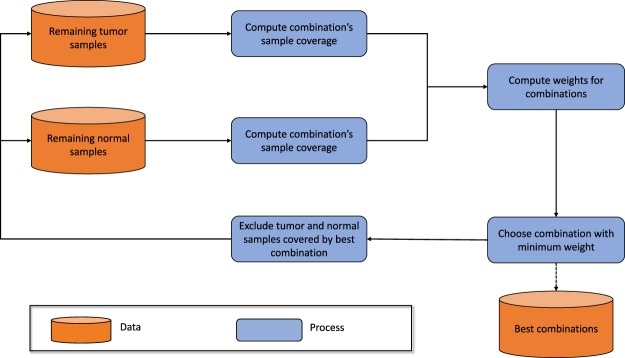
Approximation algorithm for identifying multi-hit combinations. In each iteration the algorithm selects and excludes samples that are covered by the combination with the minimum weight, until all tumor samples have been selected.

The computational complexity for finding an optimal solution to the WSC problem scales exponentially with problem size, making it computationally intractable. For the problem of finding a set of multi-hit combinations, let *G* = 20000 be the number of genes and *h* = 8 be the maximum number of hits. Then, the number of possible combinations $M={\sum }_{c=2}^{h}(\frac{G}{c})\approx 6\times {10}^{29}$. The number of possible subsets of these combinations is 2^*M*^. The optimal solution would be a subset of combinations with the minimum weight. Though a brute-force search could find the optimal solution, the size of the search-space makes the task computationally impossible. However, many approximate algorithms have been developed and analyzed for solving set cover and weighted set cover problems. We use the approximation algorithm illustrated in Fig. 9. The algorithm iteratively performs the following steps until all tumor samples have been selected:

1. Compute weights for each possible combination of genes using only the unselected samples (initially all samples)
2. Greedily choose the combination with the lowest weight
3. Select all samples containing this combination and exclude from subsequent iterations.

The computational complexity for this algorithm is O(*NM*), where *N* is the number of tumor samples and *M* is the number of possible multi-hit combinations, compared to 2^*M*^ for the brute force algorithm. Even with this approximation, the computational complexity of O(4 × 10^31^) for the number of samples *N* = 200 is still impractical with currently available computational technology. Therefore, to be able to find a solution within available computational resource we limit the number of hits to two. For *h* = 2, computational complexity is O(4 × 10^10^). In a future study we will optimize and parallelize the algorithm to make it practical to identify more than two hits.

## Data and Source

Data and source can be found at the following bitbucket repository: (https://bitbucket.org/sajal000/multihit-combinations).

## Supplementary information

Supplementary Material
