# Author Correction: Differentiating between cancer and normal tissue samples using multi-hit combinations of genetic mutations

**DOI:** 10.1038/s41598-019-53310-2

**Published:** 2019-12-09

**Authors:** Sajal Dash, Nicholas A. Kinney, Robin T. Varghese, Harold R. Garner, Wu-chun Feng, Ramu Anandakrishnan

**Affiliations:** 1 Department of Computer Science, Virginia Tech, Blacksburg, VA USA; 2 Biomedical Sciences, Edward Via College of Osteopathic Medicine, Blacksburg, VA USA; 3 Department of Electrical and Computer Engineering, Virginia Tech, Blacksburg, VA USA; 4 Gibbs Cancer Center and Research Institute, Spartanburg, SC USA

Correction to: *Scientific Reports* 10.1038/s41598-018-37835-6, published online 30 January 2019

The original version of this Article omitted an affiliation for Nicholas A. Kinney, Robin T. Varghese, Harold R. Garner and Ramu Anandakrishnan. The correct affiliations for Nicholas A. Kinney, Robin T. Varghese, Harold R. Garner and Ramu Anandakrishnan are listed below:

Biomedical Sciences, Edward Via College of Osteopathic Medicine, Blacksburg, VA, USA

Gibbs Cancer Center and Research Institute, Spartanburg, SC, USA

This error has now been corrected in the HTML and PDF versions of the Article.

