# Supplementary material for: Differentiating between cancer and normal tissue samples using multi-hit combinations of genetic mutations
Source: Sci Rep. 2019 Jan 30;9:1005. doi: 10.1038/s41598-018-37835-6 (PMC6353925; doi:10.1038/s41598-018-37835-6)
Supplement: Supplementary file 1 — Supplementary Material [file 41598_2018_37835_MOESM1_ESM.pdf]

Differentiating between cancer and normal tissue samples using multi-hit combinations of genetic mutations

(Supplementary Material)

Sajal Dash, Nicholas Kinney, Robin Varghese, Harold Garner, Wu-chun Feng, and Ramu Anandakrishnan

November 27, 2018

A Supplementary Material

A.1 Identifying somatic variants using MuTect2 and VEP: command parameters

Preliminary step of our approach is to identify meaningful somatic variations. We identify somatic variations by comparing tumor and normal tissue samples with blood-derived normal samples using MuTect2.

We use the following command for germline variant calling.

```
java -jar GenomeAnalysisTK.jar
     -T MuTect2 -R GRCh38.d1.vd1.fa
     -I:tumor tissue.bam -I:normal blood-normal.bam -o output.vcf
     --disable_auto_index_creation_and_locking_when_reading_rods
     --max_alt_alleles_in_normal_count 2
     --contamination_fraction_to_filter 0.02 --dbsnp dbsnp.vcf
     --cosmic cosmic.vcf 279
```

Once the variants are identified, we compute the effect of these variants using Variant Effect Predictor(VEP). We used the following VEP command for effect prediction:

```
perl variant_effect_predictor.pl
     -i input.vcf -o output.vep --fasta GRCh38-directory --nostats
```

A.2 Algorithm and Data Structure

Input and Data Structure We first prepare the following data structures from the tumor tissue samples and normal tissue samples.

Data	Description
$\mathsf{G}_{selected}$	a list of selected genes. $List < Gene >$.
$TumorCoverage$	a dictionary object mapping any gene to a set of tumor samples it covers. $Dict < Gene, Set < TumorSample >>$.
$NormalCoverage$	a dictionary object mapping any gene to a set of normal samples it covers. $Dict < Gene, Set < NormalSample >>$.
$TumorSamples$	a set of all tumor tissue samples. $Set < TumorSamples >$
$NormalSamples$	a set of all normal tissue samples. $Set < NormalSamples >$
$CandidateCombination$	$G_{selected} \times G_{selected}$, a set of all candidate Combinations.

A.3 Robustness of our algorithm across sets of partitions

We partitioned the data in three different ways, and the average classification performance in each case is comparable. Table S1 shows the result when we run our algorithm on the second partition.

A.4 Identified Combinations for 17 Cancer Types

Table S2-S18 show identified 2-hit combinations for 17 cancer types using the first partition.

Algorithm 1 Identifying 2-hit combinations

Require: $\mathsf{G}_{selected}$, $TumorCoverage$, $NormalCoverage$, $TumorSamples$, $NormalSamples$, $CandidateCombinations$

Initialize $C \leftarrow \phi$

$CoveredTumorSamples \leftarrow \phi$

$CoveredNormalSamples \leftarrow \phi$

$\mathbb{C}$

while $|coveredTumorSamples| \neq |TumorSamples|$ **do**

  $weights \leftarrow \phi$

  for $combination \in CandidateCombinations$ **do**

    $g_1, g_2 \leftarrow extract(combination)$

    $Cov_T(g_1) \leftarrow TumorCoverage.getSamples(g_1)$

    $Cov_T(g_2) \leftarrow TumorCoverage.getSamples(g_2)$

    $Cov_T(combination) \leftarrow Cov_T(g_1) \cap Cov_T(g_2) - CoveredTumorSamples$

    $Cov_N(g_1) \leftarrow NormalCoverage.getSamples(g_1)$

    $Cov_N(g_2) \leftarrow NormalCoverage.getSamples(g_2)$

    $Cov_N(combination) \leftarrow Cov_N(g_1) \cap Cov_N(g_2) - CoveredNormalSamples$

    $weight(combination) \leftarrow f(Cov_N(combination), Cov_T(combination))$

    $weights[combination] \leftarrow weight(combination)$

  end for

  $bestCombination \leftarrow argmin\{weights\}$

  Update $CoveredTumorSamples$ and $CoveredNormalSamples$ using $bestCombination$

  $\mathbb{C} \leftarrow \mathbb{C} \cup \{bestCombination\}$

  $CandidateCombinations \leftarrow CandidateCombinations - bestCombination$

end while

return $\mathbb{C}$

			Discovery Set								Validation Set									
			Tumor Samples				Normal Samples				Tumor Samples					Normal Samples				
Cancer Type	#Hits	#Combinations	True Positives	False Negatives	Total	Sensitivity	True Negatives	False Positives	Total	Specificity	True Positives	False Negatives	Total	Sensitivity	95% Confidence Interval	True Negatives	False Positives	Total	Specificity	95% Confidence Interval
BLCA	2	17	283	0	283	1.00	239	1	240	1.00	78	7	85	0.92	83-96%	78	15	93	0.84	74-90%
BRCA	2	7	674	0	674	1.00	234	6	240	0.97	235	2	237	0.99	96-99%	82	11	93	0.88	79-93%
CESC	2	9	209	0	209	1.00	240	0	240	1.00	57	8	65	0.88	77-94%	82	11	93	0.88	79-93%
COAD	2	10	287	0	287	1.00	239	1	240	1.00	93	5	98	0.95	88-98%	89	4	93	0.96	89-98%
GBM	2	10	252	0	252	1.00	240	0	240	1.00	71	8	79	0.90	81-95%	88	5	93	0.95	87-98%
HNSC	2	12	360	0	360	1.00	238	2	240	0.99	101	9	110	0.92	85-96%	87	6	93	0.94	86-97%
KIRP	2	10	181	0	181	1.00	239	1	240	1.00	44	3	47	0.94	82-98%	84	9	93	0.90	82-95%
LGG	2	11	360	0	360	1.00	236	4	240	0.98	112	7	119	0.94	88-97%	85	8	93	0.91	83-96%
LIHC	2	8	223	0	223	1.00	239	1	240	1.00	83	6	89	0.93	85-97%	85	8	93	0.91	83-96%
LUAD	2	12	310	0	310	1.00	239	1	240	1.00	93	6	99	0.94	87-97%	85	8	93	0.91	83-96%
LUSC	2	11	228	0	228	1.00	239	1	240	1.00	65	12	77	0.84	74-91%	89	4	93	0.96	89-98%
OV	2	9	231	0	231	1.00	240	0	240	1.00	83	3	86	0.97	90-99%	90	3	93	0.97	90-99%
PRAD	2	21	321	0	321	1.00	239	1	240	1.00	87	13	100	0.87	78-92%	72	21	93	0.77	67-85%
SARC	2	5	151	0	151	1.00	240	0	240	1.00	66	2	68	0.97	89-99%	93	0	93	1.00	96-100%
STAD	2	17	297	0	297	1.00	239	1	240	1.00	84	7	91	0.92	84-96%	77	16	93	0.83	73-89%
THCA	2	16	323	0	323	1.00	239	1	240	1.00	92	6	98	0.94	87-97%	83	10	93	0.89	81-94%
UCEC	2	6	360	0	360	1.00	234	6	240	0.97	135	0	135	1.00	97-100%	87	6	93	0.94	86-97%
Total	2	191	5050	0	5050	1.00	4053	27	4080	0.99	1579	104	1683	0.94	92-94%	1436	145	1581	0.91	89-92%

Table S1: Result for 2-hit combinations

Combination	Gene1	Gene2	#Samples Covered	Fraction Coverage
1	ENSG00000205277	ENSG00000184956	781	0.857299670692
2	ENSG00000149531	ENSG00000211896	347	0.380900109769
3	ENSG00000219481	ENSG00000173213	305	0.334796926454
4	ENSG00000185567	ENSG00000090512	81	0.0889132821076
5	ENSG00000170471	ENSG00000205869	79	0.0867178924259
6	ENSG00000178104	ENSG00000275113	47	0.0515916575192
7	ENSG00000149531	ENSG00000084731	29	0.0318331503842
8	ENSG00000137210	ENSG00000198888	8	0.00878155872667

Table S2: Sample coverage by combinations for BRCA

Combination	Gene1	Gene2	#Samples Covered	Fraction Coverage
1	ENSG00000172199	ENSG00000173213	214	0.646525679758
2	ENSG00000149531	ENSG00000211896	116	0.350453172205
3	ENSG00000149531	ENSG00000161905	109	0.329305135952
4	ENSG00000127481	ENSG00000158445	103	0.311178247734
5	ENSG00000237541	ENSG00000171862	91	0.274924471299
6	ENSG00000177731	ENSG00000124092	88	0.26586102719
7	ENSG00000198601	ENSG00000100151	41	0.123867069486
8	ENSG00000149531	ENSG00000050438	41	0.123867069486
9	ENSG00000125498	ENSG00000166272	18	0.0543806646526
10	ENSG00000241322	ENSG00000176302	6	0.0181268882175

Table S3: Sample coverage by combinations for GBM

Combination	Gene1	Gene2	#Samples Covered	Fraction Coverage
1	ENSG00000205277	ENSG00000149531	334	0.674747474747
2	ENSG00000184956	ENSG00000173213	282	0.569696969697
3	ENSG00000196126	ENSG00000171862	224	0.452525252525
4	ENSG00000205277	ENSG00000278662	188	0.379797979798
5	ENSG00000171862	ENSG00000211896	172	0.347474747475
6	ENSG00000141510	ENSG00000174501	159	0.321212121212
7	ENSG00000079841	ENSG00000205277	79	0.159595959596
8	ENSG00000198128	ENSG00000102890	22	0.0444444444444
9	ENSG00000197887	ENSG00000161031	19	0.0383838383838
10	ENSG00000243073	ENSG00000196460	10	0.020202020202

Table S4: Sample coverage by combinations for UCEC

Combination	Gene1	Gene2	#Samples Covered	Fraction Coverage
1	ENSG00000184956	ENSG00000173213	192	0.521739130435
2	ENSG00000169862	ENSG00000149531	175	0.475543478261
3	ENSG00000186409	ENSG00000139687	35	0.0951086956522
4	ENSG00000213928	ENSG00000163435	24	0.0652173913043
5	ENSG00000204479	ENSG00000124762	24	0.0652173913043
6	ENSG00000169862	ENSG00000119720	24	0.0652173913043
7	ENSG00000141510	ENSG00000171502	23	0.0625
8	ENSG00000109758	ENSG00000171936	21	0.0570652173913
9	ENSG00000099917	ENSG00000116044	16	0.0434782608696
10	ENSG00000147050	ENSG00000108840	15	0.0407608695652
11	ENSG00000240864	ENSG00000196498	13	0.0353260869565
12	ENSG00000171680	ENSG00000071626	11	0.0298913043478
13	ENSG00000153933	ENSG00000244482	11	0.0298913043478
14	ENSG00000158290	ENSG00000089041	9	0.0244565217391
15	ENSG00000156650	ENSG00000139910	9	0.0244565217391
16	ENSG00000163959	ENSG00000153815	6	0.0163043478261
17	ENSG00000205356	ENSG00000125810	5	0.0135869565217
18	ENSG00000126262	ENSG00000166736	5	0.0135869565217

Table S5: Sample coverage by combinations for BLCA

Combination	Gene1	Gene2	#Samples Covered	Fraction Coverage
1	ENSG00000184956	ENSG00000173213	129	0.565789473684
2	ENSG00000134775	ENSG00000149531	112	0.491228070175
3	ENSG00000197915	ENSG00000227152	68	0.298245614035
4	ENSG00000149531	ENSG00000204525	66	0.289473684211
5	ENSG00000169174	ENSG00000145920	37	0.162280701754
6	ENSG00000197915	ENSG00000204661	26	0.114035087719
7	ENSG00000213516	ENSG00000175193	5	0.0219298245614
8	ENSG00000159409	ENSG00000137337	4	0.0175438596491
9	ENSG00000142798	ENSG00000180767	4	0.0175438596491
10	ENSG00000070413	ENSG00000140795	4	0.0175438596491
11	ENSG00000004866	ENSG00000178188	3	0.0131578947368

Table S6: Sample coverage by combinations for KIRP

Combination	Gene1	Gene2	#Samples Covered	Fraction Coverage
1	ENSG00000184956	ENSG00000173213	176	0.577049180328
2	ENSG00000141510	ENSG00000149531	145	0.475409836066
3	ENSG00000141510	ENSG00000130226	37	0.12131147541
4	ENSG00000141510	ENSG00000221900	32	0.104918032787
5	ENSG00000141510	ENSG00000170959	31	0.101639344262
6	ENSG00000155657	ENSG00000205246	29	0.0950819672131
7	ENSG00000133863	ENSG00000141510	29	0.0950819672131
8	ENSG00000187537	ENSG00000179603	11	0.0360655737705
9	ENSG00000127507	ENSG00000121898	8	0.0262295081967
10	ENSG00000170382	ENSG00000173531	7	0.0229508196721
11	ENSG00000143882	ENSG00000167822	5	0.016393442623
12	ENSG00000007923	ENSG00000197841	3	0.00983606557377

Table S7: Sample coverage by combinations for LUSC

Combination	Gene1	Gene2	#Samples Covered	Fraction Coverage
1	ENSG00000205277	ENSG00000149531	153	0.698630136986
2	ENSG00000184956	ENSG00000173213	137	0.625570776256
3	ENSG00000205277	ENSG00000197978	87	0.397260273973
4	ENSG00000145506	ENSG00000205277	68	0.310502283105
5	ENSG00000169047	ENSG00000273976	26	0.118721461187
6	ENSG00000173662	ENSG00000006377	11	0.0502283105023

Table S8: Sample coverage by combinations for SARC

Combination	Gene1	Gene2	#Samples Covered	Fraction Coverage
1	ENSG00000184956	ENSG00000173213	188	0.686131386861
2	ENSG00000205277	ENSG00000149531	168	0.613138686131
3	ENSG00000205277	ENSG00000278662	128	0.467153284672
4	ENSG00000204525	ENSG00000149531	77	0.28102189781
5	ENSG00000205277	ENSG00000157423	62	0.226277372263
6	ENSG00000197915	ENSG00000227152	46	0.167883211679
7	ENSG00000279804	ENSG00000131951	12	0.043795620438
8	ENSG00000004455	ENSG00000178199	7	0.0255474452555
9	ENSG00000237541	ENSG00000153391	6	0.021897810219

Table S9: Sample coverage by combinations for CESC

Combination	Gene1	Gene2	#Samples Covered	Fraction Coverage
1	ENSG00000149531	ENSG00000134775	197	0.63141025641
2	ENSG00000227152	ENSG00000184956	137	0.439102564103
3	ENSG00000186844	ENSG00000134775	125	0.400641025641
4	ENSG00000177212	ENSG00000173213	120	0.384615384615
5	ENSG00000149531	ENSG00000188162	92	0.294871794872
6	ENSG00000147234	ENSG00000145920	77	0.246794871795
7	ENSG00000141510	ENSG00000130558	34	0.108974358974
8	ENSG00000198128	ENSG00000171368	13	0.0416666666667
9	ENSG00000171680	ENSG00000204310	5	0.0160256410256

Table S10: Sample coverage by combinations for LIHC

Combination	Gene1	Gene2	#Samples Covered	Fraction Coverage
1	ENSG00000184956	ENSG00000173213	252	0.616136919315
2	ENSG00000169862	ENSG00000149531	205	0.501222493888
3	ENSG00000198216	ENSG00000133703	81	0.19804400978
4	ENSG00000227152	ENSG00000010438	70	0.171149144254
5	ENSG00000116147	ENSG00000141510	63	0.154034229829
6	ENSG00000172765	ENSG00000118046	36	0.0880195599022
7	ENSG00000187741	ENSG00000081842	21	0.0513447432763
8	ENSG00000146648	ENSG00000140323	17	0.041564792176
9	ENSG00000204479	ENSG00000179593	16	0.039119804401
10	ENSG00000181396	ENSG00000156650	15	0.0366748166259
11	ENSG00000171680	ENSG00000185640	10	0.0244498777506
12	ENSG00000184677	ENSG00000165370	9	0.0220048899756
13	ENSG00000146830	ENSG00000272514	7	0.0171149144254

Table S11: Sample coverage by combinations for LUAD

Combination	Gene1	Gene2	#Samples Covered	Fraction Coverage
1	ENSG00000138413	ENSG00000184956	375	0.782881002088
2	ENSG00000184956	ENSG00000173213	294	0.613778705637
3	ENSG00000149531	ENSG00000158865	115	0.240083507307
4	ENSG00000179912	ENSG00000149531	101	0.210855949896
5	ENSG00000173826	ENSG00000177468	7	0.0146137787056
6	ENSG00000167395	ENSG00000144791	6	0.0125260960334
7	ENSG00000130244	ENSG00000112984	6	0.0125260960334
8	ENSG00000144381	ENSG00000204516	5	0.0104384133612
9	ENSG00000134184	ENSG00000122257	5	0.0104384133612

Table S12: Sample coverage by combinations for LGG

Combination	Gene1	Gene2	#Samples Covered	Fraction Coverage
1	ENSG00000184956	ENSG00000173213	308	0.655319148936
2	ENSG00000141510	ENSG00000211896	195	0.414893617021
3	ENSG00000149531	ENSG00000204525	92	0.195744680851
4	ENSG00000214324	ENSG00000055609	83	0.176595744681
5	ENSG00000146555	ENSG00000149531	64	0.136170212766
6	ENSG00000276644	ENSG00000141510	21	0.0446808510638
7	ENSG00000154222	ENSG00000099957	18	0.0382978723404
8	ENSG00000204442	ENSG00000063169	16	0.0340425531915
9	ENSG00000065526	ENSG00000021645	15	0.031914893617
10	ENSG00000146112	ENSG00000166343	12	0.0255319148936
11	ENSG00000197429	ENSG00000154175	11	0.0234042553191
12	ENSG00000198793	ENSG00000179588	4	0.00851063829787
13	ENSG00000117148	ENSG00000211967	4	0.00851063829787

Table S13: Sample coverage by combinations for HNSC

Combination	Gene1	Gene2	#Samples Covered	Fraction Coverage
1	ENSG00000184956	ENSG00000173213	221	0.569587628866
2	ENSG00000149531	ENSG00000204525	89	0.229381443299
3	ENSG00000163283	ENSG00000149531	84	0.216494845361
4	ENSG00000141510	ENSG00000177548	39	0.100515463918
5	ENSG00000110046	ENSG00000171936	39	0.100515463918
6	ENSG00000162927	ENSG00000141510	36	0.0927835051546
7	ENSG00000116251	ENSG00000198216	36	0.0927835051546
8	ENSG00000163629	ENSG00000141510	32	0.0824742268041
9	ENSG00000234745	ENSG00000039068	29	0.0747422680412
10	ENSG00000116251	ENSG00000198929	24	0.0618556701031
11	ENSG00000168702	ENSG00000120963	20	0.0515463917526
12	ENSG00000159650	ENSG00000211896	15	0.0386597938144
13	ENSG00000153201	ENSG00000100151	14	0.0360824742268
14	ENSG00000196126	ENSG00000158488	12	0.0309278350515
15	ENSG00000124466	ENSG00000185177	11	0.0283505154639
16	ENSG00000167548	ENSG00000131203	8	0.020618556701
17	ENSG00000211721	ENSG00000203933	7	0.0180412371134
18	ENSG00000184677	ENSG00000184814	3	0.00773195876289
19	ENSG00000116350	ENSG00000197245	3	0.00773195876289

Table S14: Sample coverage by combinations for STAD

Combination	Gene1	Gene2	#Samples Covered	Fraction Coverage
1	ENSG00000133056	ENSG00000158445	209	0.659305993691
2	ENSG00000141510	ENSG00000149531	173	0.545741324921
3	ENSG00000204501	ENSG00000173213	128	0.403785488959
4	ENSG00000141510	ENSG00000065534	90	0.283911671924
5	ENSG00000153820	ENSG00000141510	72	0.227129337539
6	ENSG00000155657	ENSG00000133193	71	0.223974763407
7	ENSG00000141298	ENSG00000133112	45	0.141955835962
8	ENSG00000080031	ENSG00000077782	32	0.10094637224

Table S15: Sample coverage by combinations for OV

Combination	Gene1	Gene2	#Samples Covered	Fraction Coverage
1	ENSG00000184956	ENSG00000173213	256	0.608076009501
2	ENSG00000169862	ENSG00000149531	185	0.439429928741
3	ENSG00000163283	ENSG00000149531	102	0.242280285036
4	ENSG00000038358	ENSG00000157764	92	0.218527315914
5	ENSG00000157764	ENSG00000100290	70	0.166270783848
6	ENSG00000157764	ENSG00000170369	68	0.161520190024
7	ENSG00000157764	ENSG00000186818	33	0.0783847980998
8	ENSG00000104974	ENSG00000213281	27	0.0641330166271
9	ENSG00000175216	ENSG00000211896	23	0.0546318289786
10	ENSG00000204479	ENSG00000205246	16	0.0380047505938
11	ENSG00000118777	ENSG00000100626	11	0.0261282660333
12	ENSG00000113649	ENSG00000186395	8	0.0190023752969
13	ENSG00000137492	ENSG00000155034	3	0.00712589073634

Table S16: Sample coverage by combinations for THCA

Combination	Gene1	Gene2	#Samples Covered	Fraction Coverage
1	ENSG00000184956	ENSG00000173213	247	0.586698337292
2	ENSG00000169862	ENSG00000149531	203	0.482185273159
3	ENSG00000163283	ENSG00000149531	90	0.21377672209
4	ENSG00000211896	ENSG00000168096	37	0.0878859857482
5	ENSG00000213516	ENSG00000121067	31	0.0736342042755
6	ENSG00000211896	ENSG00000135341	26	0.061757719715
7	ENSG00000100401	ENSG00000008988	25	0.0593824228029
8	ENSG00000154358	ENSG00000112559	19	0.0451306413302
9	ENSG00000187545	ENSG00000227152	17	0.0403800475059
10	ENSG00000196498	ENSG00000159625	14	0.0332541567696
11	ENSG00000204442	ENSG00000221923	12	0.0285035629454
12	ENSG00000196539	ENSG00000205445	12	0.0285035629454
13	ENSG00000198502	ENSG00000197595	11	0.0261282660333
14	ENSG00000177548	ENSG00000180104	8	0.0190023752969
15	ENSG00000152661	ENSG00000043355	8	0.0190023752969
16	ENSG00000196187	ENSG00000152086	7	0.0166270783848
17	ENSG00000198128	ENSG00000162009	6	0.0142517814727
18	ENSG00000134545	ENSG00000185519	6	0.0142517814727
19	ENSG00000116721	ENSG00000142546	4	0.00950118764846
20	ENSG00000143226	ENSG00000099889	2	0.00475059382423

Table S17: Sample coverage by combinations for PRAD

Combination	Gene1	Gene2	#Samples Covered	Fraction Coverage
1	ENSG00000134982	ENSG00000149531	281	0.72987012987
2	ENSG00000184956	ENSG00000173213	214	0.555844155844
3	ENSG00000149531	ENSG00000180329	158	0.41038961039
4	ENSG00000120314	ENSG00000157423	60	0.155844155844
5	ENSG00000184634	ENSG00000198786	42	0.109090909091
6	ENSG00000176542	ENSG00000100151	39	0.101298701299
7	ENSG00000204130	ENSG00000188766	24	0.0623376623377
8	ENSG00000154330	ENSG00000000971	20	0.0519480519481
9	ENSG00000162620	ENSG00000110074	10	0.025974025974

Table S18: Sample coverage by combinations for COAD

A.5 Correlation between Genes within Combinations

We investigate whether the sample coverages of genes within a combination are correlated in normal samples to determine if we may be identifying passenger mutations as part of the combinations. Figure S1 shows $-\log_{10} p$ against Pearson's correlation coefficients, where p is the p-value. We find no evidence of the genes within combinations being significantly correlated.

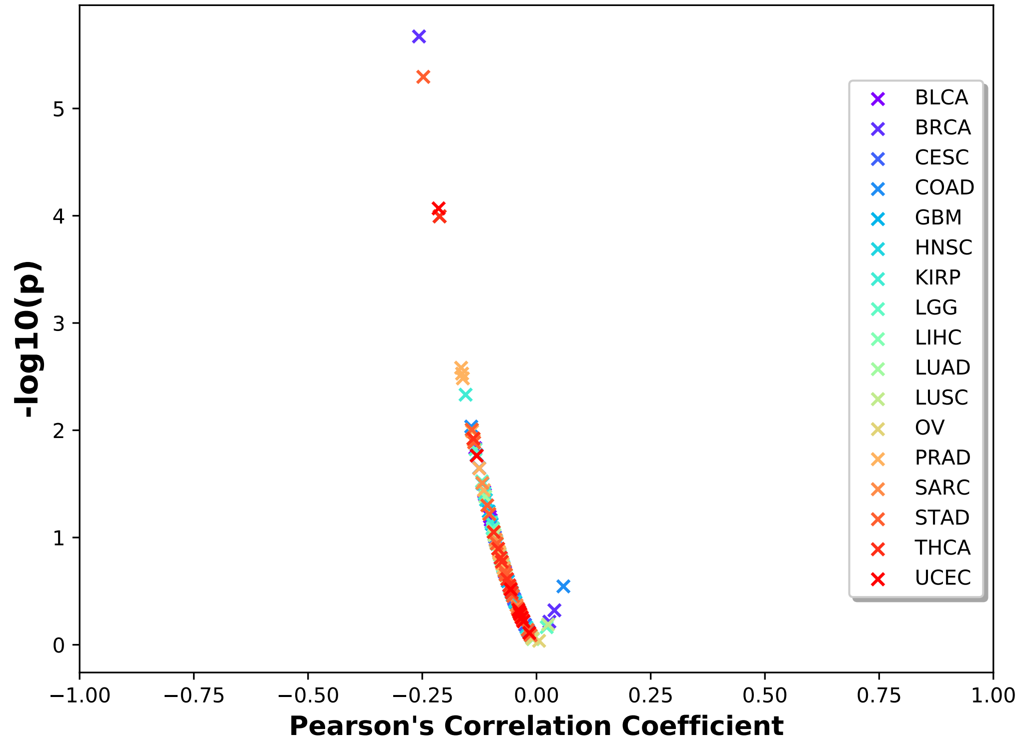

Figure S1: $-\log_{10} p$ vs Pearson correlation coefficient plot for all pairs of genes in all combinations identified by our method using the normal samples, where p is the p-value. Since no pair has a p-value < 0.005 (or $-\log_{10} p \geq 2.301$) and absolute Pearson correlation coefficient greater than 0.50, none of the combinations appear to have correlated genes.

A.6 Coverage of Samples by Identified Combinations

Figure S3 shows fractions of tumor samples covered by identified combinations. Most samples are covered by the top combinations, while a very small number of samples require a large number of combinations. Figure S4 shows that this distribution is similar for different ways of partitioning data.

Figure S5 shows that many samples can be covered by more than one combinations. These overlapping combinations might constitute more than two hits.

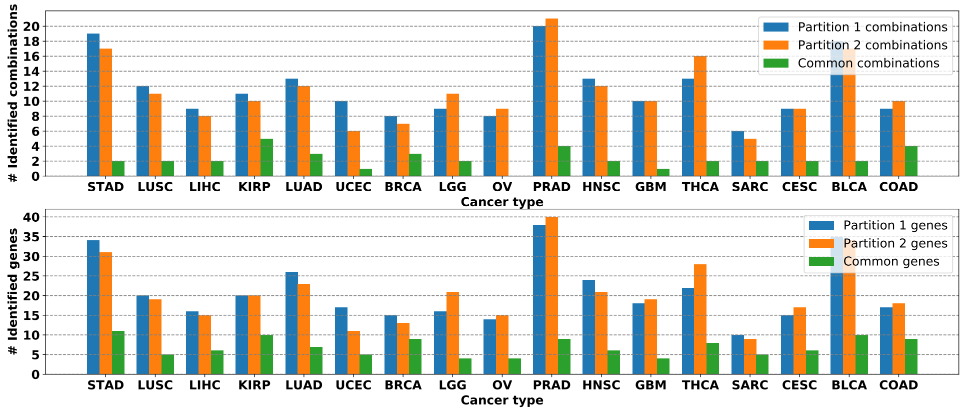

Figure S2: Identified genes and combinations shared between two sets of partitions. $(1-5)$ combinations are shared between two sets and $(4-10)$ genes are shared between two sets.

A.7 Distinguishing between driver and passenger mutations

Recatome superpathways associated with the genes in the top three 2-hit combinations are shown in Tables S19-S22.

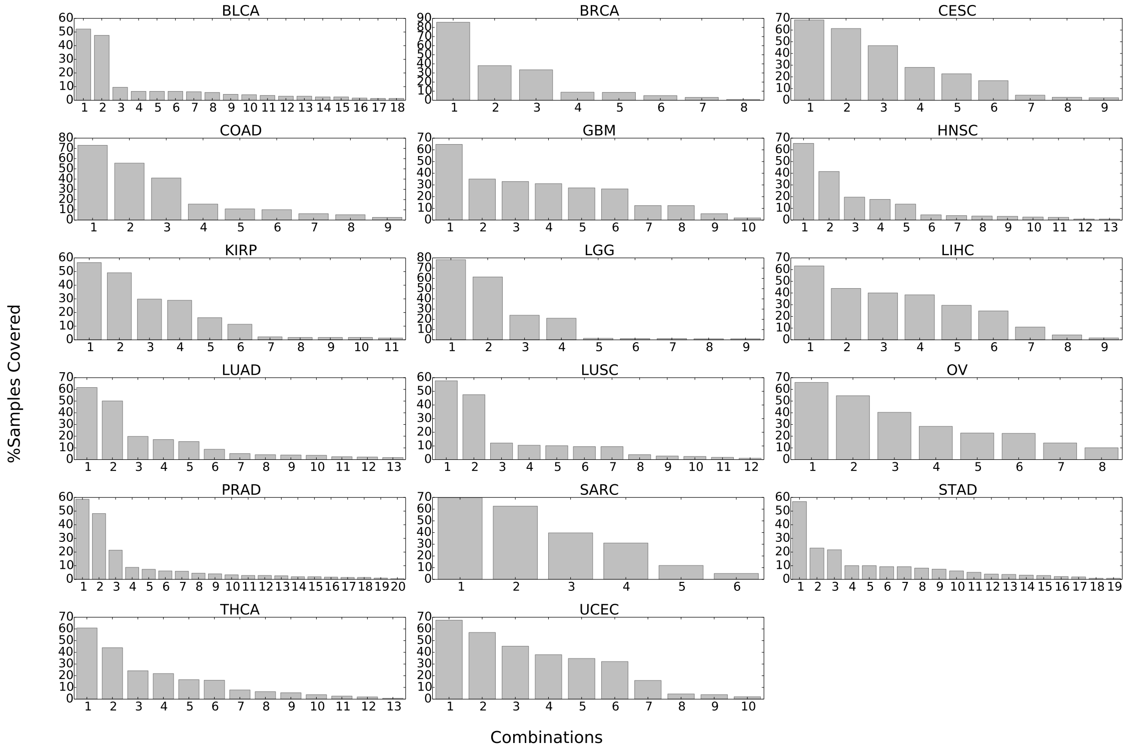

Figure S3: Occurrence of 2-hit combinations identified in tumor samples, for the seventeen cancer types considered. The top combination occurs in 65% of tumor samples, on average, while 42% of the combinations occur in less than 5% of the samples. Total percentage exceeds 100% because samples can contain multiple combinations.

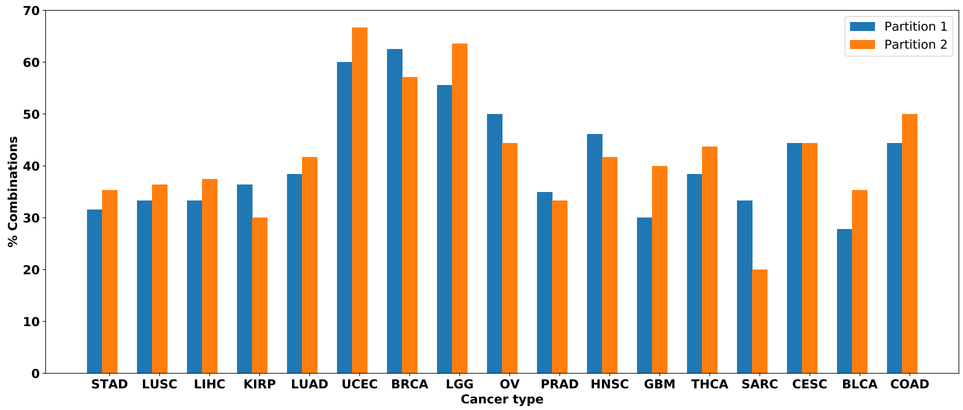

Figure S4: Percentage of identified combinations covering the last 5% of samples for the 17 cancer types. On average last 42% of the combinations occur in 5% of the tumor samples.

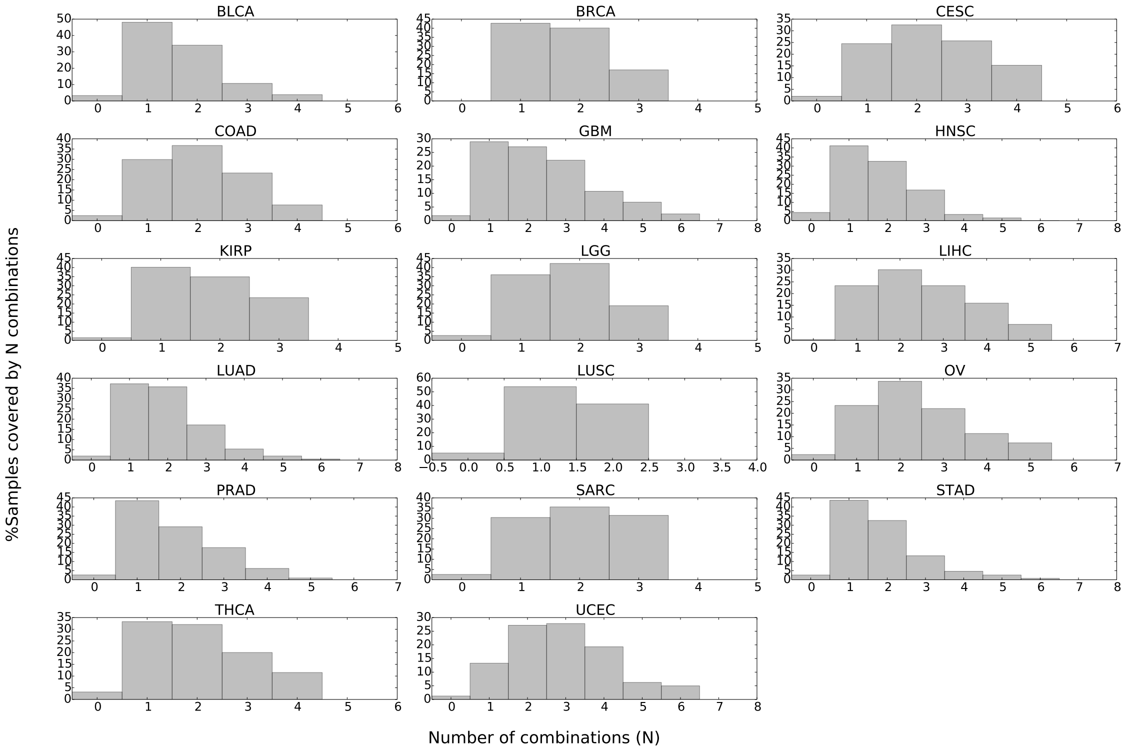

Figure S5: Distribution of overlapping combinations for seventeen cancer types. 64.5% of tumor samples contain multiple combinations, suggesting that the 2-hit combinations represent subsets of three or more hits.

Cancer	Gene	Reactome Superpathways																							
Cancer	Gene	Signal Transduction	Metabolism	Immune System	Gene Expression	Metabolism of proteins	Developmental Biology	Disease	Vesicle-mediated transport	Cell Cycle	Homeostasis	Cellular response to stress	Organelle biogenesys and maintenance	Neuronal Systems	DNA Repair	Extracellular matrix organization	Chromatin organization	Muscle contraction	Programmed Cell Death	Cell-Cell communication	DNA Replication	Circadian Clock	Reproduction	Mitophagy	Transmembrane transport of small molecules
BLCA	MUC6					X		X																	
	TUBB8P12																								
	CTNND2																								
	FRG1BP																								
	CCDC30																								
	RB1									X		X									X				
BRCA	MUC12					X		X																	
	MUC6					X		X																	
	FRG1BP																								
	IGHG1			X																					
	AHNAK2																								
	FETUB																								
CESC	MUC6					X		X																	
	TUBB8P12																								
	MUC12					X		X																	
	GOLGA6L10																								
	MUC12					X		X																	
	FRG1BP																								
COAD	APC	X				X		X											X						
	FRG1BP																								
	MUC6					X		X																	
	TUBB8P12																								
	FRG1BP																								
	CCDC43																								

Table S19: Reactome superpathways associated with genes in the top three 2-hit combinations for BLCA, BRCA, CESC and COAD.[Fabregat et. al., Nucleic Acids Research, 44(D1):D481-D487, 2016] For each cancer type, the first two genes represent the first combination, the second two genes the second combination and the third two genes the third combination.

		Reactome Superpathways																							
**Cancer**	**Gene**	Signal Transduction	Metabolism	Immune System	Gene Expression	Metabolism of proteins	Developmental Biology	Disease	Vesicle-mediated transport	Cell Cycle	Homeostasis	Cellular response to stress	Organelle biogenesys and maintenance	Neuronal Systems	DNA Repair	Extracellular matrix organization	Chromatin organization	Muscle contraction	Programmed Cell Death	Cell-Cell communication	DNA Replication	Circadian Clock	Reproduction	Mitophagy	Transmembrane transport of small molecules
GBM	OR8U1	X																							
	TUBB8P12																								
	FRG1BP																								
	ALOX15		X																						
	UBR4			X																					
	KCNB1		X											X											
HNSC	MUC6					X		X																	
	TUBB8P12																								
	TP53	X		X	X	X				X	X	X			X				X						
	IGHG1			X																					
	FRG1BP																								
	HLA-C			X																					
KIRP	MUC6					X		X																	
	TUBB8P12																								
	FHOD3																								
	FRG1BP																								
	HRNR			X																					
	OR2T7																								
LGG	IDH1		X	X				X																	
	MUC6					X		X																	
	MUC6					X		X																	
	TUBB8P12																								
	R3HDM2																								
	FRG1BP																								

Table S20: Reactome superpathways associated with genes in the top three 2-hit combinations for GBM, HNSC, KIRP, and LGG.[Fabregat et. al., Nucleic Acids Research, 44(D1):D481-D487, 2016] For each cancer type, the first two genes represent the first combination, the second two genes the second combination and the third two genes the third combination.

Cancer	Gene	Reactome Superpathways																							
		Signal Transduction	Metabolism	Immune System	Gene Expression	Metabolism of proteins	Developmental Biology	Disease	Vesicle-mediated transport	Cell Cycle	Homeostasis	Cellular response to stress	Organelle biogenesys and maintenance	Neuronal Systems	DNA Repair	Extracellular matrix organization	Chromatin organization	Muscle contraction	Programmed Cell Death	Cell-Cell communication	DNA Replication	Circadian Clock	Reproduction	Mitophagy	Transmembrane transport of small molecules
LIHC	FRG1BP																								
	FHOD3																								
	OR2T7																								
	MUC6					X		X																	
	OR2T33	X																							
	TUBB8P12																								
LUAD	MUC6					X		X																	
	TUBB8P12																								
	CTNND2																								
	FRG1BP																								
	OR2T7																								
	PRSS3		X	X																					
LUSC	MUC6					X		X																	
	TUBB8P12																								
	TP53	X		X	X	X				X	X	X			X				X						
	FRG1BP																								
	TP53	X		X	X	X				X	X	X			X				X						
	DPP6																								
OV	PIK3C2B		X																						
	KCNB1		X											X											
	TP53	X		X	X	X				X	X	X			X				X						
	FRG1BP																								
	TP53	X		X	X	X				X	X	X			X				X						
	MYLK	X																X							

Table S21: Reactome superpathways associated with genes in the top three 2-hit combinations for LIHC, LUAD, LUSC and OV.[Fabregat et. al., Nucleic Acids Research, 44(D1):D481-D487, 2016] For each cancer type, the first two genes represent the first combination, the second two genes the second combination and the third two genes the third combination.

		Reactome Superpathways																							
**Cancer**	**Gene**	Signal Transduction	Metabolism	Immune System	Gene Expression	Metabolism of proteins	Developmental Biology	Disease	Vesicle-mediated transport	Cell Cycle	Homeostasis	Cellular response to stress	Organelle biogenesys and maintenance	Neuronal Systems	DNA Repair	Extracellular matrix organization	Chromatin organization	Muscle contraction	Programmed Cell Death	Cell-Cell communication	DNA Replication	Circadian Clock	Reproduction	Mitophagy	Transmembrane transport of small molecules
PRAD	MUC6					X		X																	
	TUBB8P12																								
	CTNND2																								
	FRG1BP																								
	RBMXL1																								
	SPOP	X																							
SARC	MUC12					X		X																	
	FRG1BP																								
	MUC6					X		X																	
	TUBB8P12																								
	MUC12					X		X																	
	GOLGA6L9																								
STAD	MUC6					X		X																	
	TUBB8P12																								
	ALPP								X																
	FRG1BP																								
	ATG2A																								
	OR10H3	X																							
THCA	MUC6					X		X																	
	TUBB8P12																								
	CTNND2																								
	FRG1BP																								
	EDC4				X																				
	BRAF	X		X			X	X						X											
UCEC	MUC12					X		X																	
	FRG1BP																								
	MUC6					X		X																	
	TUBB8P12																								
	HLA-DRB1			X																					
	PTEN	X	X	X	X	X		X																	

Table S22: Reactome superpathways associated with genes in the top three 2-hit combinations for PRAD, SARC, STAD, THCA and UCEC.[Fabregat et. al., Nucleic Acids Research, 44(D1):D481-D487, 2016] For each cancer type, the first two genes represent the first combination, the second two genes the second combination and the third two genes the third combination.
